# Association between Microalbuminuria Predicting In-Stent Restenosis after Myocardial Infarction and Cellular Senescence of Endothelial Progenitor Cells

**DOI:** 10.1371/journal.pone.0123733

**Published:** 2015-04-13

**Authors:** Hisanobu Ota, Naofumi Takehara, Tatsuya Aonuma, Maki Kabara, Motoki Matsuki, Atsushi Yamauchi, Toshiharu Takeuchi, Jun-ichi Kawabe, Naoyuki Hasebe

**Affiliations:** 1 Department of Internal Medicine, Division of Cardiology, Nephrology, Pulmonology and Neurology, Asahikawa Medical University (AMU), Asahikawa, Japan; 2 Department of Cardiovascular Regeneration and Innovation, Asahikawa Medical University (AMU), Asahikawa, Japan; University of Kansas Medical Center, UNITED STATES

## Abstract

**Objective:**

Relationship between microalbuminuria and worse outcome of coronary artery disease patients is discussed, but its underlying pathophysiological mechanism remains unclear. We investigated the role of microalbuminuria to the function of endothelial progenitor cells (EPCs), that might affect to outcome of acute myocardial infarction (AMI) patients.

**Methods:**

Forty-five AMI patients were divided into two groups according to their urinary albumin excretion: normal (n = 24) and microalbuminuria (>30 mg/day, n = 21). At day-2 and day-7 after AMI onset, circulating-EPCs (CD34^+^Flk1^+^) were quantified by flow cytometry. The number of lectin-acLDL-positive cultured-EPCs immobilized on fibronectin was determined. To assess the cellular senescence of cultured-EPCs, the expression level of sirtuin-1 mRNA and the number of SA-β-gal positive cell were evaluated. Angiographic late in-stent loss after percutaneous coronary intervention (PCI) was evaluated at a six-month follow-up.

**Results:**

No significant differences in coronary risk and the extent of myocardial damage were observed between the two groups. Late in-stent loss at the six-month follow-up was significantly higher in the microalbuminuria group (normal : microalbuminuria = 0.76±0.34 : 1.18±0.57 mm, p=0.021). The number of circulating-EPCs was significantly increased in microalbuminuria group at day-7, however, improved adhesion of EPCs was observed in normal group but not in microalbuminuria group from baseline to day-7 (+3.1±8.3 : -1.3±4.4 %: p<0.05). On the other hand, in microalbuminuria group at day-7, the level of sirtuin-1 mRNA expression of cultured-EPCs was significantly decreased (7.1±8.9 : 2.5±3.7 fold, p<0.05), which was based on the negative correlation between the level of sirtuin-1 mRNA expression and the extent of microalbuminuria. The ratio of SA-β-gal-positive cells in microalbuminuria group was increased compared to that of normal group.

**Conclusions:**

Microalbuminuria in AMI patients is closely associated with functional disorder of EPCs via cellular senescence, that predicts the aggravation of coronary remodeling after PCI.

## Introduction

Microalbuminuria, defined as a subtle increase in the urinary albumin excretion (UAE), is a well-established predictor of detrimental cardiovascular events [1]. Recently, it has attracted attention as a risk factor for in-stent restenosis in coronary artery disease (CAD) patients who have undergone percutaneous coronary intervention (PCI) [2]. Furthermore, others and we have reported that microalbuminuria in patients with acute myocardial infarction (AMI) has been associated with in-hospital and long-term mortality [3–6]. However, it remains unclear whether microalbuminuria in AMI patients is correlated with the pathogenesis of coronary atherosclerosis that is based on disrupted endothelial repair.

EPCs play an important role in endogenous endothelial repair [7], particularly during the process of reendothelialisation after coronary intervention, because of their unique adhesion, migration, and endothelial regeneration properties [8–10]. Although several studies have indicated that pathophysiological actions of EPCs play a pivotal role in coronary atherosclerosis and endothelial dysfunction, the extent and direction of EPC mobilization in CAD patients continues to be debated in the literature [8,10,11]. Moreover, the relationship between functional disorder of EPCs and cardiovascular outcome has also been discussed [12–15]. A recent report showed that cellular damage by oxidative stress in metabolic syndrome (MS) is associated with telomere shortening in circulating-EPCs of CAD patients [16]. And then, Tentolouris et al. also observed that the telomere length of white blood cells in type 2 diabetes patients with microalbuminuria is shorter than that in subjects without microalbuminuria [17]. However, the pathogenic role of microalbuminuria under disrupted endothelial repair, especially under impairment of EPC function, has not been fully understood.

Here, we have assessed the pathophysiological role of microalbuminuria in the early phase of AMI with focusing on the function of EPC. It will provide novel evidence for an association between the functional disorder of EPC and microalbuminuria, those may cause adverse coronary outcomes of AMI patients.

## Methods

### Study population

This study was conducted at the Asahikawa Medical University Hospital between January 2010 and December 2011 in accordance with the Declaration of Helsinki. All patients gave written informed consent.

**Fig 1:**
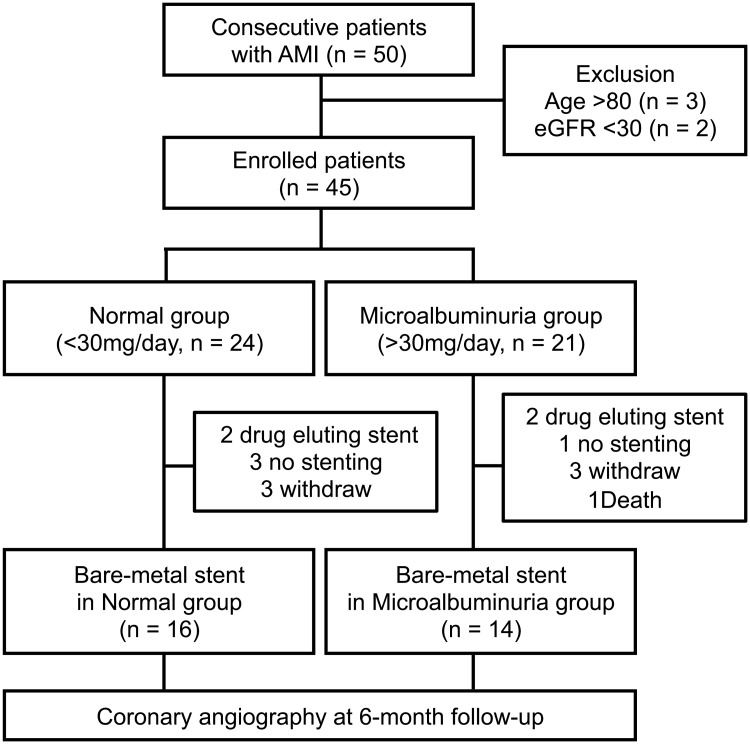
Study design.

Each of the 50 AMI patients enrolled in this study underwent emergent angiography within 24 hours of AMI diagnosis. The overall design of the study can be found in Fig 1. Exclusion criteria were 1. age over 80 years, 2. the estimated glomerular filtration rate (eGFR) <30 mL·min^-1^ per 1.73 m^2^. After emergent angiography, urine was collected from all patients over a 24-hour time period. Patients were then divided into 2 groups according to UAE: normal group (<30 mg/day) and microalbuminuria group (>30 mg/day). Primary PCI was performed with the conventional approach using a 6-French guiding catheter to use manual thrombectomy devices, thrombolytic agents, and stents. Dual antiplatelet therapy was administered to all patients and was maintained at least 1 month. Patients who received bare-metal stents were followed until angiographic reevaluation at 6 months after PCI.

### Ethics Statement

The protocol used in this study was approved by the Institutional Ethics Committee of Asahikawa Medical University.

### Definitions

The clinical parameters assessed for each patient included age, sex, smoking habit, hypertension, diabetes mellitus, dyslipidemia, body mass index, and eGFR. Initial risk stratification was performed according to the Global Registry of Acute Coronary Events Risk Score (GRACE RS). The factors were age, heart rate, systolic blood pressure, creatinine, Killip class, cardiac arrest, elevated cardiac markers, ST elevation myocardial infarction (STEMI), and interventional device (stenting and intra-aortic balloon pump). Contrast-induced nephropathy was defined as an absolute increase in serum creatinine of 0.5 mg/dL and/or a relative increase of 25% in serum creatinine. Plasma brain natriuretic peptide (BNP) levels were measured at 2 to 4 weeks after the onset of AMI. Patients underwent transthoracic echocardiographic examinations to assess left ventricular function 2 to 4 weeks after the onset of AMI.

### Coronary outcome

Angiographic findings were evaluated using a quantitative coronary angiography system on a TCS Acquisition workstation (Medcon Ltd.). The coronary events highlighted in this evaluation included: hospitalization for unstable angina with objective evidence of ischemia, coronary revascularization including target lesion revascularization (TLR), in-stent restenosis assessed by late lumen loss, stenosis diameter, and the ratio of binary restenosis defined as 50% or more lumen narrowing at follow-up coronary angiography in the culprit vessel, accounting for lesion length and stent size. TLR was defined as a repeat revascularization for a stenosis 50% or more in the target segment in the presence of ischemia. To focus on the role of microalbuminuria on the primary coronary outcome, AMI patients who had drug-eluting stents (DES) implantation were excluded because DES was previously shown to reduce coronary restenosis in comparison to bare-metal stents, leading to biased results.

### Quantification of circulating-EPCs

Peripheral blood samples were collected twice, on days 2 and 7 after admission. Day 2 samples were defined as the baseline level of circulating- and cultured-EPCs in each patient, and day 7 samples were evaluated to determine levels of mobilized circulating and cultured-EPCs associated with onset of AMI. Peripheral blood mononuclear cells (PBMNCs) were enriched using density gradient centrifugation with Lymphoprep solution (Axis-Shield Poc AS. Norway). Flow cytometer was used to identify the circulating-EPCs in PBMNCs using double-positive staining for CD34 and KDR (kinase insert domain receptor, known as vascular endothelial cell growth factor receptor 2) established as a marker of the circulating-EPC in previous reports [18–20]. Single-cell suspensions of PBMNC were stained with the following antibodies: fluorescein isothiocyanate (FITC)-conjugated mouse anti-human CD34 (BD Pharmingen), phycoerythrin (PE)-conjugated mouse anti-human KDR (Sigma), and PE- and FITC-conjugated isotype control immunoglobulins (BD Pharmingen). FACSCalibur flow cytometer (BD Biosciences, San Jose, CA) was used to evaluate the level of cellular immunostaining in each sample.

### EPC cell culture

PBMNCs were incubated for 7 days on fibronectin-coated dishes using endothelial cell growth medium MV2 supplemented with 10% fetal bovine serum, hydrocortisone, ascorbic acid, heparin sulfate, 2% penicillin/streptomycin, 50 ng/mL human recombinant VEGF, insulin-like growth factor 1, basic fibroblast growth factor, and epidermal growth factor (all material from PromoCell). To confirm the cultured cells as EPC, we fixed them with 4% paraformaldehyde and stained them using FITC-conjugated Ulex europeaus agglutinin-I (UEA-1, Sigma). Additionally, the uptake of 1,1-dioctadecyl-3,3,3,3-tetramethylindocarbocyanine (DiI)-labeled acetylated low-density lipoprotein (acLDL, Life Technologies) was monitored, as cultured-EPCs are known to be positive for both UEA-1 and acLDL. Double-positive cells that adhered to the fibronectin-coated dishes were counted in five randomly selected fields using an inverted fluorescent microscope. Nuclei were visualized using Hoechst 33342 (Lonza). To assess the adhesion ability of the cultured-EPCs, the number of adherent EPCs was compared to the initial number of PBMNCs plated.

### Quantitative reverse-transcription PCR

Total RNA was isolated from cultured-EPCs after seven days of cultivation by the phenol-chloroform method using TRIzol reagent (Invitrogen). To produce cDNA, 1000 ng of poly (A) RNA from each sample was used for reverse transcription (RT) using the SuperScript-III cDNA synthesis kit (Invitrogen) according to the manufacturer’s instructions. Quantitative RT-PCR reactions were performed using Taq-man Gene Expression primers for human sirtuin-1 (Applied Biosystems, Inc.) and Taq-man Gene Expression Master mix (Applied Biosystems, Inc.). Thermal cycling conditions consisted of 2 min at 50°C and 10 min at 95°C, followed by 40 cycles of 15 s at 95°C and 1 min at 60°C. A 7300/7500 Real-time PCR system was employed for PCR thermocycling, data collection, and analysis (Applied Biosystems Inc.). The relative fold increase of sirtuin-1 mRNA expression in AMI patients with or without microalbuminuria was calculated relative to that in age-matched healthy volunteers.

### Senescence-associated beta (β)-galactosidase assay

To assess the phenotypic senescent changes in cultured-EPCs, we stained the cells for senescence-associated beta-galactosidase (SA-β-gal; Senescence Detection Kit, BioVision, Inc.), a well-known biomarker of cellular senescence. In a 6-well plate, cells were fixed with 0.5 mL of Fixative Solution for 10 min at room temperature. After the fixed cells were washed, 0.5 mL of a staining solution mixture containing X-gal was added and incubated for 6 hours at 37°C. Cellular staining for SA-β-gal was observed using a light microscope, and positive cells were counted. Senescent cells presented a blue color.

### Statistical analyses

Categorical data in baseline patient characteristics were presented as numbers and percentages, and comparisons between groups were performed using Fisher’s exact test. Continuous data for laboratory data, cardiac function, the number of circulating-EPCs, the relative fold increase ofsirtuin-1 mRNA expression, the number of SA-β-gal-positive cells in the cultured-EPCs at day 7, and coronary outcome were presented as mean ± standard deviation and were compared using Student’s t-test or the Mann—Whitney U test. All data in these analysis which did not follow the normal distribution set were assessed by the Man-Whitney U test and their degree of variance included some outliners. The absolute change in the adhesion ability of the cultured-EPCs between baseline and day 7 after the onset of AMI was compared using the Mann-Whitney U test and Wilcoxon signed-rank tests. Spearman’s linear regression analysis was used to assess the correlation between the level of microalbuminuria and EPC function (the number of adherent EPCs and sirtuin-1 mRNA expression in the cultured-EPCs). Logistic multiple regression analysis was used for logarithm UAE, sex, age, Killip class, CIN, and smoking history as independent variables to evaluate effects on sirtuin-1 mRNA expression, considered to represent EPC function. Group differences were considered statistically significant at p <0.05. Statistical analyses were conducted using IBM SPSS version 18.0.

## Results

### Patient characteristics

Forty-five patients were eligible to participate in this study; 3 of the patients were older than 80 years and 2 had eGFR levels lower than 30 mL·min^-1^·1.73 m^-2^. According to their UAE, 24 patients were assigned to the normal group and 21 were assigned to the microalbuminuria group. The baseline characteristics of the study population are shown in Table 1. No significant differences in coronary risk factors, ratio of ST-elevated myocardial infarction (STEMI), PCI procedures, final thrombolysis in myocardial infarction (TIMI) flow, Killip class, or prescribed drugs were observed between the two groups. Additional laboratory data and cardiac function evaluations are presented in S1 Table. We observed no significant differences in the peak level of creatine kinase, creatine kinase MB isotype, and plasma BNP between the two groups. The eGFR was similar for the two groups during the first 48 hours after the onset of AMI. Transthoracic echocardiography also indicated that LV size, contractility, and diastolic function were similar between the two groups after AMI.

**Table 1:** Patient characteristics.

	Normal	Microalbuminuria	p value
	(n = 24)	(n = 21)	
Age (year)	62.8 ± 11.8	62.6 ± 11.3	0.960
Male (%)	70.1	85.7	0.296
Hypertension (%)	75.0	71.4	1.00
Diabetes (%)	33.3	33.3	1.00
Dyslipidemia (%)	83.3	90.5	0.670
Current Smoker (%)	33.3	57.1	0.140
Prior CAD (%)	12.5	19.0	0.689
Killip III, IV (%)	4.2	19.0	0.169
GRACE RS	123.7 ± 31.6	141.4 ± 48.8	0.152
Systolic BP (mm Hg)	141.5 ± 26.3	147.7 ± 31.1	0.473
Heart rate (beats/min)	77.4 ± 18.3	82.7 ± 20.7	0.371
STEMI (%)	91.7	85.7	0.652
Anterior MI (%)	58.3	42.9	0.376
Onset-Balloon (min)	450.8 ± 434.4	405.8 ± 413.5	0.724
Stent (%)	95.8	95.2	0.611
IABP (%)	0	4.8	0.463
Final TIMI flow 3 (%)	95.8	95.2	1.00
CIN (%)	4.2	23.8	0.083
Medications (%)			
β-blocker (%)	62.5	52.4	0.555
ACE-I (%)	45.8	28.6	0.356
ARB (%)	41.7	57.1	0.376
CCB (%)	33.3	28.6	0.759
Diuretic (%)	12.5	28.6	0.267
Statin (%)	87.5	76.2	0.443
Antiplatelet therapy (%)	95.8	100	1.00

CAD, coronary artery disease; GRACE RS, Global Registry of Acute Coronary Events risk score; BP, blood pressure; IABP, intra-aortic balloon pump; STEMI, ST elevation myocardial infarction; MI, myocardial infarction; TIMI, Thrombolysis in Myocardial Infarction; CIN, contrast induced nephropathy.

### Relationship between microalbuminuria and coronary outcome

Coronary outcomes were assessed for 30 AMI patients with bare-metal stent implantation at 6 month after primary PCI because 15 patients were not included in the follow-up analysis (6 withdraw, 4 no stenting, 4 DES implantation, 1 early death) (Table 2). At baseline, no significant difference in the size of the implanted stent and the distribution of infarct-related coronary arteries was seen between the two groups. Although, in the microalbuminuria group, target lesion revascularization was undergone in two patients with binary restenosis with myocardial ischemia (exercise induced ischemia detected by radio-isotope scintigraphy) and one patient with binary restenosis without myocardial ischemia was treated with a drug therapy in the microalbuminuria group, we did not find a significant different in the onset of angina or in the extent of target lesion revascularization between the two groups. However, late lumen loss, an indicator of in-stent restenosis, was significantly increased in the microalbuminuria group (1.18±0.57 mm) relative to the normal group (0.76±0.34 mm; p = 0.021). Furthermore, the microalbuminuria group tended to show increased extent and frequency of stenosis.

**Table 2:** Coronary outcomes.

	Normal	Microalbuminuria	p value
	(n = 16)	(n = 14)	
Infarction related artery			0.529
LAD (%)	62.5	42.9	
Cx (%)	12.5	14.3	
RCA (%)	25.0	42.9	
Stent size			
Diameter (mm)	3.04 ± 0.31	3.14 ± 0.42	0.460
Length	24.38 ± 13.78	21.14 ± 6.04	0.424
Immediately after procedure			
Minimum luminal diameter (mm)	2.79 ± 0.43	3.03 ± 0.53	0.173
At 6-month follow-up			
Hospitalization for unstable angina	0	0	1.00
Minimum luminal diameter (mm)	2.02 ± 0.50	1.86 ± 0.65	0.436
Reference lesion diameter (mm)	2.79 ± 0.50	2.97 ± 0.64	0.383
Late lumen loss (mm)	0.76 ± 0.34	1.18 ± 0.57	0.021
Diameter stenosis (%)	27.44 ± 12.58	37.63 ± 17.70	0.077
Binary restenosis rate (%)	0	21.4	0.090
Target lesion revascularization (%)	0	14.3	0.209

### Mobilization and function of EPCs

**Fig 2:**
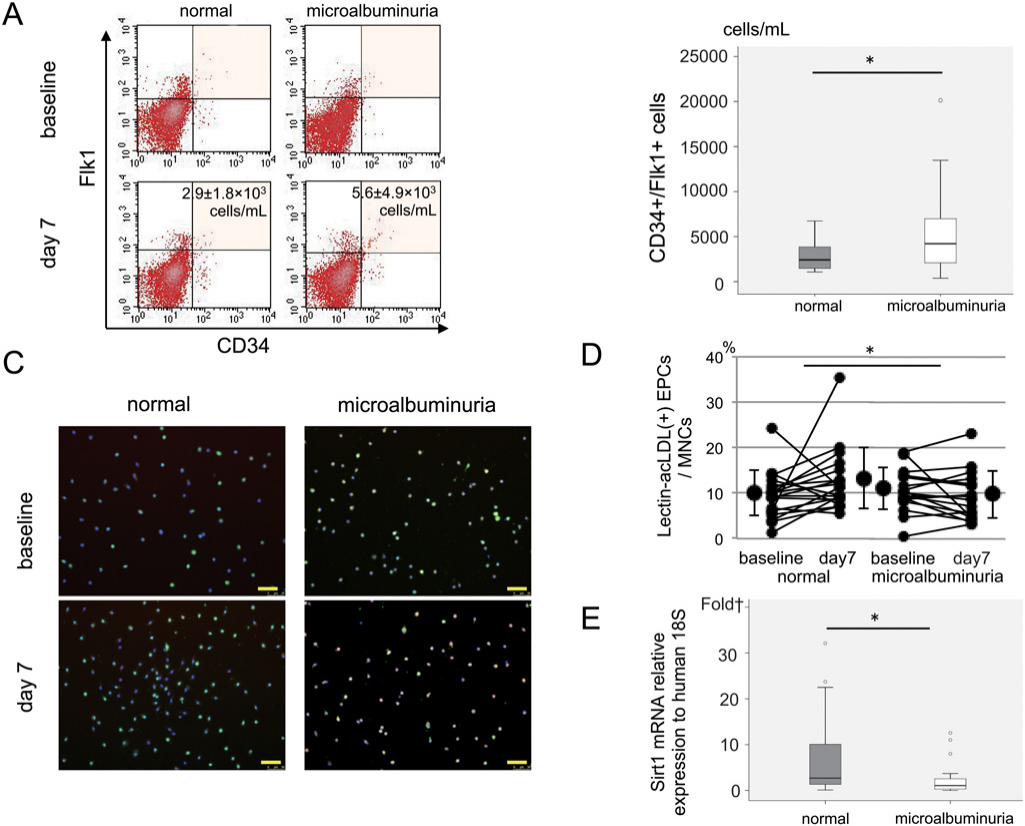
Circulating and cultured EPCs from the peripheral blood of AMI patients. A: FACS analysis of circulating EPCs. B: Number of circulating EPCs. C: Immunochemistry of cultured EPCs. Lectin (green), acLDL (red), merged double-positive for lectin and acLDL (orange), Scale bar = 50 μm. D: Adhesive ability of the cultured EPCs E: Sirtuin1 mRNA expression in the cultured EPCs *p<0.05 for AMI patients without albuminuria (normal) compared to AMI patients with microalbuminuria (microalbuminuria). In the box plot, the central box shows the first quartile to the third quartile, which means the interquartile range (IQR). The line in the box shows the median, and the line above and below the box shows 1.5 × IQR or maximum and minimum (if there is no outliner), respectively. The circles (1.5 × IQR or more) and small rectangles (3 × IQR or more) show outliners. Scale bar = 50μm

To clarify the mechanism of increasing in-stent restenosis in the microalbuminuria group, we evaluated the mobilization and function of EPCs after AMI. The number of circulating-EPCs in the PBMNC samples from AMI patients was determined using FACS analysis in the sub-acute (day-7) phases of AMI (Fig 2A). Although no significant differences were observed in the number of circulating-EPCs at baseline between the two groups, the number of circulating-EPCs at day-7 was significantly higher in the microalbuminuria group (5580±4941 cells/mL compared to 2921±1765 cells/mL for the normal group; p<0.05) (Fig 2B). In contrast, the absolute change of adhesive ability of EPCs in the normal group from baseline to day-7 was significantly larger than that in the microalbuminuria group (normal group vs. microalbuminuria group = +3.1±8.3% vs. -1.3±4.4%; p<0.05) (Fig 2C and 2D). The expression level of Sirtuin-1 mRNA in the cultured-EPCs did not differ between the two groups at baseline (3.0±4.2 vs. 3.9±5.6 fold = the normal group vs the microalbunuria group; p = ns). However, at day-7, although the expression level of Sirtuin-1 mRNA in the cultured-EPCs of the normal group enhanced from the baseline, that of the microalbuminuria group decreased. Therefore, the expression level of Sirtuin-1 mRNA in the cultured-EPCs of microalbuminuria group (2.5±3.7 fold) was significantly lower than that of the normal group at day-7 (7.1±8.9 fold; p<0.05) (Fig 2E).

### Correlation between microalbuminuria and EPC function

**Fig 3:**
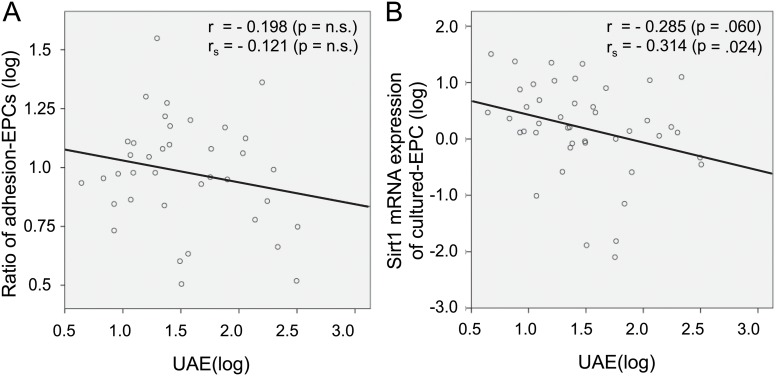
Correlation between UAE and EPC function in linear regression analysis.

To investigate the role of microalbuminuria to EPC function, we assessed the correlation between the level of microalbuminuria and EPC function represented by cell adhesion (the ratio of cultured-EPCs) and cellular senescence (Sirtuin-1 mRNA expression) in linear regression analysis among enrolled patients. There was no significant correlation between the level of microalbuminuria (UAE) and the ratio of adherent EPCs at day-7 (Fig 3A), however, the level of Sirtuin-1 mRNA expression was negatively associated with the extent of UAE (Fig 3B). Furthermore, when we assessed the effect of confounding factors on sirtuin-1 mRNA expression of cultured-EPCs in the enrolled patients, UAE was the only independent predictor among age, sex, current smoking, killip level, and contrast-induced nephropathy in a logistic multivariate analysis (Table 3).

**Table 3:** Logistic multivariate analysis to Sirtuin1 mRNA expression as a marker for senescence of EPC.

	Odds ratio (95% confidence interval)	p value
Log UAE	0.167 (0.031–0.886)	0.036
Sex	0.362 (0.114–1.151)	0.085
Killip III, IV	0.117 (0.007–1.839)	0.127
CIN	2.165 (0.253–18.507)	0.481
Age	1.020 (0.953–1.091)	0.575
Current Smoker	1.573 (0.318–7.786)	0.579

UAE, urinary albumin excretion; CIN, contrast induced nephropathy.

### Senescence of cultured-EPCs

**Fig 4:**
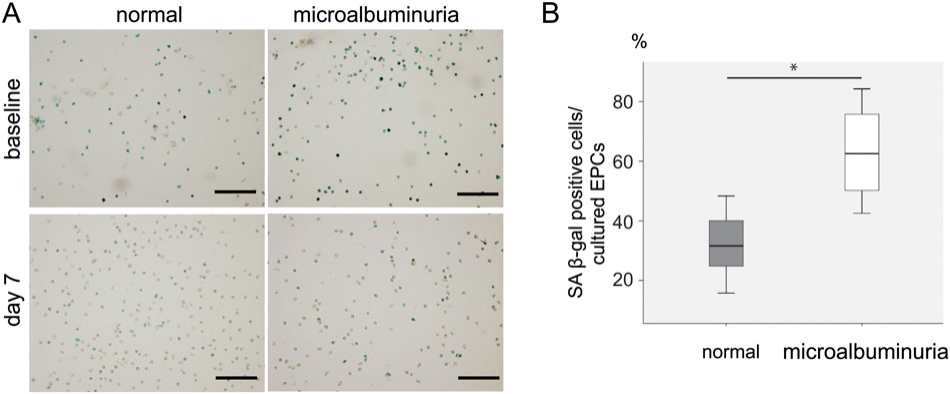
Cellular senescence of cultured EPCs of AMI patients. A: SA-β-gal staining (shown in blue) of cultured EPCs at baseline and day-7. B: Percentage of SA-β-gal-positive cells. *p<0.05 for AMI patients without albuminuria (normal) compared to AMI patients with microalbuminuria(microalbuminuria). †p<0.01 comparing the day-7 samples in AMI patients without albuminuria (normal). In the box plot, the central box shows the first quartile to the third quartile, which means the interquartile range (IQR). The line in the box shows the median, and the line above and below the box shows maximum and minimum, respectively. Scale bar = 100μm

To assess the senescence of the EPCs associated with decreasing level of Sirtuin-1 mRNA expression, we performed a SA-β-gal assay to cultured-EPCs as a supplemental study using 10 of the AMI patients; six of these patients were normal group (60.3±12.6 years old), whereas 4 were microalbuminuria group (72.3±10.0 years old). No significant difference in age distribution was observed between two groups. At baseline (day-2), the ratio of SA-β-gal-positive cells in cultured-EPCs from AMI patients did not differ between two groups (56.1±17.1% vs. 62.1±7.4%; p = ns) (Fig 4A). However, cultured-EPCs in microalbuminuria group had a significantly higher ratio of senescent cells than those in normal group at day-7 (normal group vs. microalbuminuria group = 32.0±11.8% vs. 63.5±17.5%; p<0.05) (Fig 4A and 4B).

## Discussion

In the present study, we demonstrated in AMI patients that 1) late in-stent lumen loss was significantly increased in the microalbuminuria group at six months after PCI, 2) the number of circulating-EPCs with a reduced ability to bind to fibronectin was increased in the microalbuminuria group compared to the normal group in the acute phase of MI, and 3) the level of sirtuin-1 mRNA expression of cultured-EPCs in the microalbuminuria group was lower than that of the normal group. These findings indicate that microalbuminuria of AMI patients predicts worse coronary outcome and may be associated with EPC dysfunction (senescence).

It is actually unclear whether the number of mobilized EPCs in CAD patients is altered in patients at cardiovascular risk, despite important roles played by circulating-EPCs in CAD patients [21]. In general, levels of circulating-EPCs in AMI patients have been observed to increase from onset to 7 days after AMI, but the fluctuation of circulating-EPCs is not so striking in patients with fewer lethal cardiovascular risk factors [22, 23]. For example, in this study, the level of hsCRP was significantly higher in the microalbuminuria group. Though we could not show the direct correlation between hsCRP and EPC mobilization, hsCRP is known to be a circulating inflammatory marker that reflects systemic vascular damage, and considered to be an independent cardiovascular risk factor as well as microalbuminuria [24, 25]. Recently, Ling et al. showed that the number of circulating-EPCs presented a greater increase in AMI patients with diabetes mellitus (DM) than in AMI patients without DM [13]. Meanwhile, in HT patients with microalbuminuria, Huang et al. reported that the number of circulating-EPCs was reduced, whereas the number of circulating apoptotic endothelial cells (positive for CD31/annexinV) was increased, implying a function related to EPC viability [26]. In the present study, we also found an increased number of circulating-EPCs in the microalbuminuria group than in the normal group, despite the lack of difference in the number of circulating-EPCs at baseline between two groups. Therefore, our results also suggest that microalbuminuria is a pivotal risk factor that affects the mobilization of EPCs from bone marrow in AMI patients.

The deterioration of EPCs in AMI patients is closely associated with not only mobilization but also cellular function (e.g. adhesion, regeneration, and senescence). For example, decreased expression of phospho-Akt, phospho-eNOS, and HIF activity in EPCs of AMI patients with DM seems to be involved in EPC dysfunction [13]. Satoh et al. observed in the telomere shortening and increased levels of 8-hydroxyl deoxyguanosine in EPCs of AMI patients with MS, suggesting that oxidative stress plays a role in telomere shortening and likely stimulates mobilization of EPCs during the acute phase of　MI to compensate for EPC dysfunction [16]. Paschalaki et al. recently reported that the EPC dysfunction of smokers and patients with chronic obstructed pulmonary disease caused by DNA damage and cellular senescence was negatively correlated with expression of sirtuin-1 [27]. They also indicated that EPC dysfunction resulted in a failure to recover blood flow in an in vivo mouse model of angiogenesis. In this study, we found in microalbuminuria group that the adhesion ability of EPCs was reduced at day-7 despite the increased mobilization of EPCs from baseline to day-7, and also observed in the decreased level of sirtuin-1 mRNA expression and the EPC senescence indicated as the lack of improvement in SA-β-gal staining at day 7. Under the pathological conditions that result in microalbuminuria, acute myocardial ischemia may actualize latent EPC dysfunction and increase the mobilization both viable and senescence EPC to support angiogenesis. Recently, Wang et al reported that NAMPT-NAD^+^-Sirtuin-1 (not Sirtuin 2–7) cascade improved post-ischemic vascular repair by modulating Notch signaling in mouse EPCs [28]. Enhanced-expression of NAMPT protein by ischemic insult (peak at Day-7) upregulated the expression of Sirtuin-1 in EPCs, and improved the migration of EPCs. This beneficial effect against the ischemic injury was reduced in EPCs with the silencing of Sirtuin-1 through the activation of Notch-signaling. The malfunction of Sirtuin-1 cascade in EPC of patients might generate microalbuminuria in AMI via some mechanisms, for example, unstabilization of glomerular endothelial function. Moreover, the persistent senescence of EPCs may induce in-stent restenosis in microalbuminuria group, which contributes the impairment of re-endothelialization after vascular injury. Although many investigators have examined the relationship between the number of circulating-EPCs and in-stent restenosis [8,10,29,30], to our knowledge, this is the first study to provide novel pathophysiological evidence to explain the worse outcomes in AMI patient with microalbuminuria.

This study showed the increased tendency of contrast induced nephropathy (CIN) in the microalbuminuria group. Our result was correspondent with these data, and impaired endothelial function and inflammation may increase vascular permeability and glomerular albumin leakage as one of the mechanisms of microalbuminuria. However, another potential mechanism of CIN in microalbuminuria group is considered to be a direct injury to tubular epithelium and microalbuminuria may be developed with impaired tubular reabsorption of albumin.

We have several limitations in this study. The first limitation was stent delivery. In the present study, we excluded 4 DES patients from follow-up study. Bare-metal stent have been commonly used in acute myocardial infarction, but in evidence on the safety accumulated so far, drug-eluting stents have been also routinely used in acute myocardial infarction with severe coronary atherosclerosis in culprit lesion to reduce the binary restenosis, target lesion revascularization, and late lumen loss. However, the outcome of PCI patients is affected by not only the restenosis of the target lesion but also the progression of neo-intima formation in another lesions. Impaired function of EPC with cellular senescence might not be able to prevent the progression of another vessel damage by the ramification injury of coronary intervention. Therefore, even in DES era, the evidence of this study may become a milestone to improve the worse outcome of the AMI patient with microalbuminuria and EPC dysfunction. And the second limitation of this study was the small sample size for evaluation of the correlation among the 3 factors: microalbuminuria, EPC function, and coronary late loss. Although our study could not directly show evidence that microalbuminuria causes impairment of EPC function, the correlation between UAE (microalbuminuria) and sirtuin-1 mRNA expression (EPC function) was significantly shown in all enrolled patients of this study, despite the presence of many confounding factors affecting sirtuin-1 mRNA expression in cultured-EPCs (Table 3). Furthermore, others and we had reported a relationship between microalbuminuria and worsened outcome of AMI patients, and this study especially demonstrated that the late lumen loss of AMI patients was closely associated with microalbuminuria. To provide the first step towards a better understanding between the EPC function of microalbuminuria group and in-stent restenosis, future work should investigate the relationship among microalbuminuria, AMI, and EPC function using a large-scale clinical trial to confirm our observations.

In conclusion, our results suggest that microalbuminuria of AMI patients is associated with EPC dysfunction, which aggravates coronary remodeling. Its underlying pathophysiological mechanism may involve, at least in part, a relationship between microalbuminuria and EPC senescence.

## Supporting Information

S1 Table Laboratory data and cardiac function. (DOCX)
