# Supplementary material for: Association between Microalbuminuria Predicting In-Stent Restenosis after Myocardial Infarction and Cellular Senescence of Endothelial Progenitor Cells
Source: PLoS One. 2015 Apr 13;10(4):e0123733. doi: 10.1371/journal.pone.0123733 (PMC4395282; doi:10.1371/journal.pone.0123733)
Supplement: S1 Table — (DOCX) [file pone.0123733.s001.docx]

Supporting Information S1 Table . Laboratory data and cardiac function

|  | Normal | Microalbuminuria | p value |
| --- | --- | --- | --- |
|  | (n = 24) | (n = 21) |  |
| WBC (× 10^3^/L) | 9.5 ± 2.8 | 10.6 ± 2.9 | 0.233 |
| Hemoglobin (g/dL) | 14.2 ± 2.4 | 14.6 ± 1.9 | 0.516 |
| Platelet (× 10^4^/L) | 22.3 ± 7.0 | 21.1 ± 5.1 | 0.541 |
| Glucose (mg/dL) | 148.5 ± 55.4 | 190.2 ± 115.9 | 0.154 |
| T-Chol (mg/dL) | 203.5 ± 34.6 | 204.0 ± 39.9 | 0.966 |
| eGFR (mL/min/1.73m^2^) | 73.2 ± 16.7 | 74.8 ± 22.6 | 0.788 |
| UAE (mg/day) | 15.1 ± 7.4 | 112.1 ± 88.6 | <.001 |
| hsCRP (mg/L) | 1.8 ± 3.2 | 5.0 ± 6.0 | 0.043 |
| Peak CK (× 10^3^IU/L) | 3.2 ± 2.1 | 3.2 ± 2.0 | 0.895 |
| Peak CK-MB (IU/L) | 304.3 ± 206.0 | 291.4 ± 176.2 | 0.824 |
| BNP (pg/mL) | 128.3 ± 101.6 | 112.7 ± 86.7 | 0.591 |
| Echocardiography |  |  |  |
| LV EDVI (mL/m2) | 53.7 ± 19.4 | 56.4 ± 19.0 | 0.646 |
| LV ESVI (mL/m2) | 25.1 ± 13.3 | 25.2 ± 10.8 | 0.975 |
| LV EF (%) | 54.9 ± 10.2 | 56.3 ± 7.1 | 0.600 |
| E (cm/sec) | 58.8 ± 20.7 | 55.7 ± 15.5 | 0.580 |
| A (cm/sec) | 65.8 ± 18.2 | 70.1 ± 18.9 | 0.363 |
| DcT (msec) | 182.7 ± 40.6 | 202.9 ± 51.1 | 0.152 |
| E/A | 0.99 ± 0.54 | 0.80 ± 0.13 | 0.102 |
| E/e’ | 11.3 ± 5.3 | 11.7 ± 3.9 | 0.796 |

WBC, white blood cell; T-Chol, total cholesterol; eGFR, estimated glomerular filtration rate; hsCRP, high sensitivity c-reactive protein; UAE, urinary albumin excretion; CK, creatinine kinase; BNP, brain natriuretic peptide.LV, left ventricular; EDVI, end-diastolic volume index; ESVI, end-systolic volume index; EF, ejection fraction; DcT, deceleration time.
